# Do preoperative fear avoidance model factors predict outcomes after lumbar disc herniation surgery? A systematic review

**DOI:** 10.1186/2045-709X-21-40

**Published:** 2013-11-18

**Authors:** Faris A Alodaibi, Kate I Minick, Julie M Fritz

**Affiliations:** 1 Health Rehabilitation Sciences Department, King Saud University, Riyadh, Saudi Arabia; 2 Department of Physical Therapy, University of Utah, Salt Lake City, UT, USA

**Keywords:** Fear-avoidance, Catastrophizing, Depression, Pain, Disability, Lumbar disc herniation

## Abstract

**Background:**

Lumbar disc herniation (LDH) surgery is usually recommended when conservative treatments fail to manage patients’ symptoms. However, many patients undergoing LDH surgery continue to report pain and disability. Preoperative psychological factors have shown to be predictive for postoperative outcomes. Our aim was to systematically review studies that prospectively examined the prognostic value of factors in the Fear Avoidance Model (FAM), including back pain, leg pain, catastrophizing, anxiety, fear-avoidance, depression, physical activity and disability, to predict postoperative outcomes in patients undergoing LDH surgery.

**Methods:**

We performed a systematic literature review of prospective studies that measured any FAM factors preoperatively to predict postoperative outcomes for patients undergoing LDH surgery. Our search databases included PubMed, CINAHL, and PsycINFO. We assessed the quality of each included study using a certain quality assessment list. Degree of agreement between reviewers on quality assessment was examined. Results related to FAM factors in the included studies were summarized.

**Results:**

Thirteen prospective studies met our inclusion criteria. Most studies were considered high quality. Heterogeneity was present between the included studies in many aspects. The most common FAM factors examinered were baseline pain, disability and depression. In, general, depression, fear-avoidance behaviors, passive pain coping, and anxiety FAM factors appeared to have negative influence on LDH surgical outcome. Baseline back pain and leg pain appeared to have differing prognostic value on LDH surgical outcomes.

**Conclusions:**

FAM factors seem to influence LDH surgical outcomes. Patients with high levels of depression, anxiety and fear-avoidance behaviors are more likely to have poor outcomes following LDH surgery. Conversely, high levels of leg pain, but not back pain seem to be predictor for favorable LDH surgery outcome. More research is needed to determine the exact role of FAM factors on LDH surgical outcome and the value for screening for these factors.

## Background

Lumbar discectomy or surgery to remove a lumbar disk herniation (LDH) compressing a nerve root is usually recommended when 6 to 8 weeks of conservative treatments fail to relieve sciatica symptoms. In the U.S., Medicare spending (in 2003) on discectomy/laminectomy surgeries exceeded 300 million dollars [1]. However, long-term surgical outcomes for more than one third of the patients undergoing discectomy were not satisfactory and more than one quarter continue to have significant residual pain after surgery [2,3]. Additionally, reoperation rates after lumbar discectomy range from 9% to 25% [3-5]. Careful selection and screening for prognostic factors is crucial to minimize substantial costs and unfavorable outcomes.

The Fear Avoidance Model (FAM) is composed of physical, cognitive, emotional, and behavioral constructs that have been found to be associated with future disability and pain persistence [6,7]. Several studies have found these factors predict the development of low back pain (LBP) as well as the transition and maintenance of chronic LBP [8-11]. According to the FAM, an individual with catastrophic cognitions about pain tends to interpret a pain experience as threatening to his/her health. This cognitive interpretation, in turn, triggers fear and avoidance of activities that are perceived by the patient to be related to pain. As the patient continues with such maladaptive beliefs and behaviors, disuse, disability, and depression may subsequently develop.

Examining the prognostic value of FAM factors has been mostly conducted in nonoperative and nonspecific LBP populations. Additional studies have fonud that preoperative biopsychosocial factors are, in general, predictive of postsurgical outcomes [12-15]. Nevertheless, studies that measured specific preoperative FAM factors to predict LDH surgical-outcomes are scarce. Additionally, evidence about which FAM factor are most predictive of LDH postsurgical outcomes is not yet clear. Therefore, our aim in this systematic review was to identify prospective studies that have included FAM factors before discectomy surgery to predict LDH postoperative outcomes, including pain and disability, and to identify which FAM measures have prognostic value for surgical outcomes in this population.

## Methods

We performed a systematic search using relevant databases including Medline (PubMed 1980–2012), PsycINFO (EBSCO 1980–2012), and CINAHL (EBSCO 1981–2012). We manually searched related reviews and studies’ reference lists. We used a wide range of keywords to ensure including most of the studies that pertained to our aim. In our search, we combined keywords related to back pain and/or sciatica, disc herniation, surgery to remove herniation, and FAM factors with “AND” search query (detailed search’s keywords is displayed below). We included studies that fit our inclusion criteria (Table 1).

### The systematic review search strategy (keywords)

Search performed using the following keywords strategy:All the steps were then combined with “AND”.

1. Studies examining LBP identified using: low back pain, backache, lumbago, “lumbar radiculopathy”, sciatica, back pain, dorsalgia, and “leg pain,” combined with “OR” statements.

2. Studies related to the disc herniation identified using: Disc, bulge, protrusion, prolapse, herniation, slipped, combined with “OR” statements.

3. Studies that included patients undergoing LDH surgery identified using: surgery, operation, operative, preoperative, postoperative, postsurgical, discectomy, microdiscectomy, combined with “OR” statements.

4. Studies that included FAM predictors identified using: pain, catastrophizing, catastrophising, affectivity, sensitivity, anxiety, vigilance, hypervigilance, attention, fear, kinesiophobia, avoidance, depression, physical activity, disuse, deconditioning, disability, and coping, combined with “OR” statements.

**Table 1:** The systematic review inclusion criteria

**Inclusion criteria**	
1. Prospective design (i.e., observational study or a secondary analysis of a randomized control trial-RCT).	4. LDH had to be confirmed by clinical diagnostic test (MRI, CT, or myelography) or by operative findings (i.e., bulging/protrusion, prolapse, extrusion, or sequestration).
2. Study should have included any of the FAM factors preoperatively (back pain, leg pain, pain catastrophizing, pain coping, fear, avoidance, anxiety, functional disability, depression, or physical activity) to predict postoperative pain, disability, or return to work outcome (or a composite measure that included anyone of the aforementioned outcomes).	5. All preoperative FAM measures have been taken within 6 weeks prior to surgery.
3. All included patients were scheduled to undergo surgery to remove LDH causing symptoms related to sciatica (i.e., either discectomy or microdiscectomy).	6. Follow-up outcome measures were taken at least 3 months after surgery.
	7. Did not include patients with other diagnoses (e.g., stenosis, spondylolistesis, or arthritis).

We included only full report studies with enough description of the methods to allow our review. We did not have language or sample size restrictions. However, because surgical procedures have changed, we limited our search to studies published after 1980.

### Search and extraction procedure

Two independent reviewers (FA and KM) conducted the review search. The initial step included screening titles and abstracts followed by screening the full text of potentially eligible studies. Disagreements between reviewers about a study’s eligibility were resolved by consensus in a meeting with a third reviewer (JF). Once an article was selected for inclusion in the review, the pertinent data were extracted by the lead author. Each included study was assessed for the association between the included preoperative FAM variables and the postoperative outcomes. We examined primarily multivariate analyses that were used to test FAM predictors. We considered preoperative predictors to be measures of FAM factors that were related to back pain, leg pain, pain catastrophizing, pain coping, fear, avoidance, anxiety, functional disability, depression, or physical activity. Postoperative outcomes that we considered were pain intensity, functional disability, and ability to return to work (or a composite measure that included any one of the aforementioned outcomes).

### Quality assessment

The same two reviewers (FA and KM) assessed each included study’s methodological quality using a list of criteria (Table 2) to evaluate prognostic studies as reported by Hayden et al. [16] Agreement between reviewers on each quality assessment criterion for each study was examined using weighted Kappa statistics (with 95% CI). Each criterion was given a score of two if it was satisfied in the study, one if it was partially achieved, and zero if the criterion was not achieved or was not clear. The total possible score for each study based on these 11 criteria was 22. Studies that scored 18 or higher (>80%) were considered high quality studies, and studies with a score less than 18 were considered low quality studies.

**Table 2:** The quality assessment criteria

**Domain**	**Criteria**
Sample	1- Source of the sample were clearly defined
	2- Enough description of the sample
Prognostic variables	3- Clear definition and description of the used prognostic factor
	4- Measured appropriately (reliable and valid)
Follow-up	5- Completeness rate (>80%)
	6- Adequate description of the completeness
Outcome	7- Clear definition and description of the used outcomes
	8- Measured appropriately (reliable and valid)
Analysis	9- Enough description of the analysis
	10- Appropriate analysis
Confounding	11- Account for potential confounders with appropriate analysis

## Results

**Figure 1:**
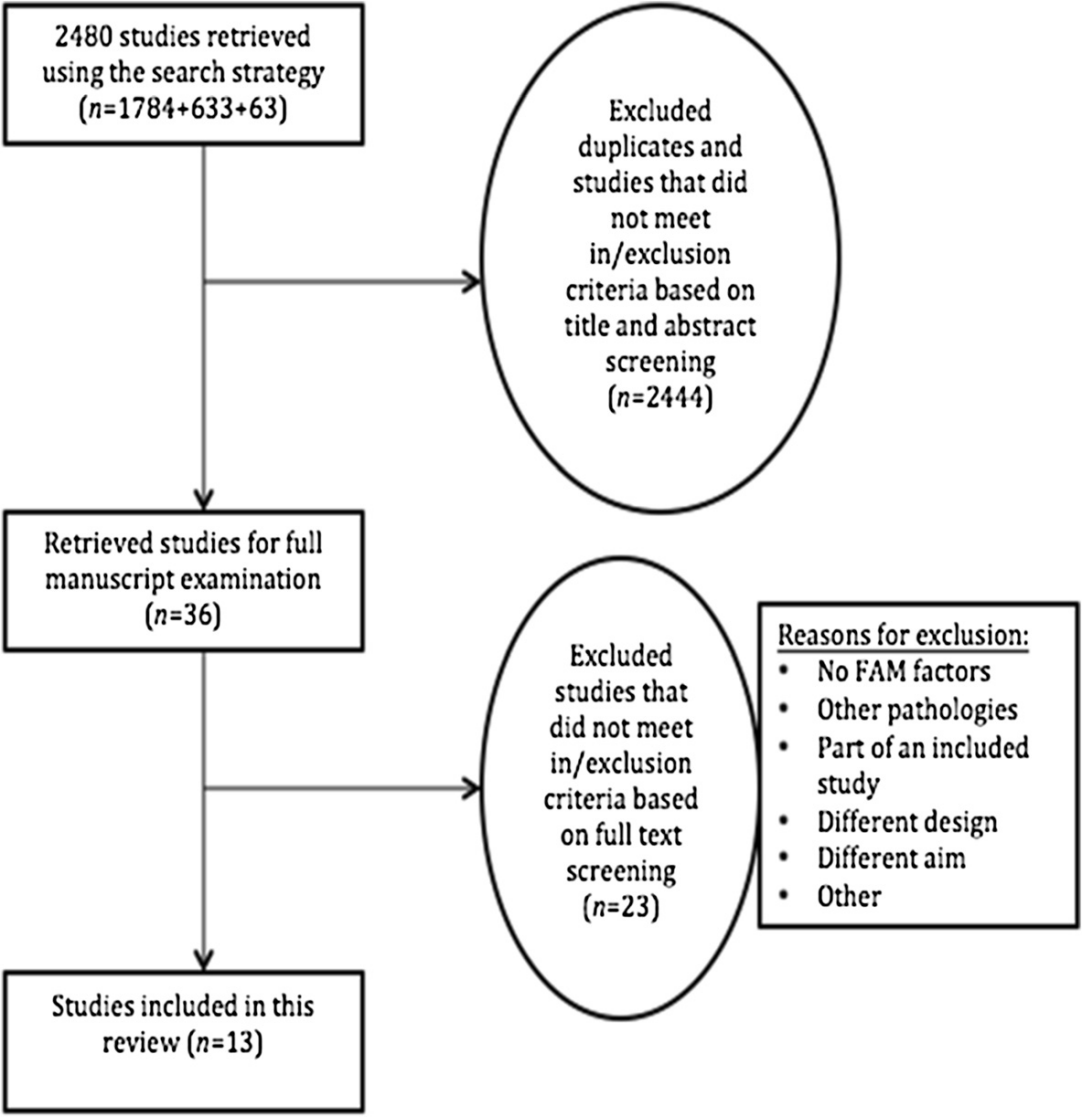
Search process flow diagram.

Out of 2480 citations, we screened the full text of 36 potentially eligible studies. Thirteen studies met the inclusion criteria and were included [17-29]. A flow diagram, illustrating the review process is presented in Figure 1. A summary table of the characteristics of each included study is shown in (Additional file 1: Table S4) and (Additional file 2: Table S5). The most common reasons for excluding studies after a full text screening were: the study had a different aim and did not use appropriate analyses, the study design was not prospective, or the study was part of another included study.

### Heterogeneity of the included studies

Heterogeneity was present between included studies in terms of which FAM predictor measures were evaluated, the outcome measures used, the length of the follow-up periods, and the analyses used to test predictors and control for potential confounding variables. Therefore, it was not appropriate to conduct a meta-analysis. Instead, we reviewed and summarized the results of the included studies.

### Description of the included studies

All included studies aimed primarily to examine the predictive value of one or more FAM factors for LDH surgical outcomes. All of the studies included subjects with LDH diagnosis who were candidates for surgery to remove the herniated disc. All of the included studies took place in Europe. Sample sizes in the included studies ranged from 46 [21] to 342 [19] and follow-up (FU) rates were over 80% in all studies except one that did not report the FU rate [22]. The FU periods ranged from 6 months to 7 years in two studies [20,27]. The surgical procedures performed were either discectomy or microdiscectomy. Although most studies used regression analyses to test prediction models of outcomes, two studies used discriminant analysis [18,19] and one used cluster analysis [23]. Six studies included an adjustment for baseline leg pain, back pain, or functional disability in their prediction models [19,21,24,27-29], while five studies had an adjustment for other variables [17,20,22,25,26]. One study was originally a randomized clinical trial that did not find a significant difference between two rehabilitation programs [26]. Another study used data that were prospectively collected on consecutive patients undergoing discectomy [29].

### Quality assessment (QA)

Out of the 143 total QA items evaluated across the included studies, the two reviewers agreed on 122 items (85.3%). Overall interrater agreement of the QA between the two raters was good [30] kappa = 0.66 (*p*<.001), 95% CI (0.53, 0.79). Interrater agreement on the individual QA criteria ranged from fair to very good (Kappa values, 0.20-1.00). A QA table of the included studies is attached below (Table 3). The QA score for the included studies ranged from 13 to 21 (out of 22). Four studies that scored lower than 18 (80%) on the QA were considered low quality studies [17,18,22,23].

**Table 3:** Quality assessment table

**Study**	**Sample**		**Prognostic factors**		**Follow-up**	
	**Source of sample clearly defined**	**Enough description of the sample**	**Clear definition/description of the used prognostic factor**	**Measured appropriately (reliability, validity)**	**Completeness rate (>80%)**	**Adequate description of completeness**
Fulde et al. 1995 [18]	P	P	Y	Y	Y	N
A. Junge et al. 1995 [19]	P	Y	Y	Y	Y	Y
Schade et al. 1999 [21]	P	Y	Y	Y	Y	N
V. GRAVER et al. 1999 [20]	Y	Y	Y	Y	Y	Y
Kohlboek et al. 2004 [23]	Y	Y	P	Y	Y	N
L. Arpino et al. 2004 [22]	Y	P	Y	Y	N	N
Den Boer et al. 2006 [24]	P	Y	Y	Y	Y	Y
Silverplats et al. 2010 [27]	P	Y	Y	Y	Y	N
JOHANSSON et al. 2010 (A) [26]	Y	Y	Y	Y	Y	Y
D’Angelo et al. 2010 [25]	Y	Y	Y	Y	Y	P
Kleinstueck et al. 2011 [29]	Y	Y	Y	P	Y	N
Chaichana et al. 2011 [28]	P	Y	Y	Y	Y	Y
Sorensen and Mors 1989 [17]	Y	P	P	Y	Y	Y
**Study**	**Ouxtcome**		**Analysis**			**Score out of (22) Y=2, P=1, N=0**
	**Clear definition/description of the used outcome**	**Measured appropriately (reliability, validity)**	**Enough description**	**Appropriate analysis**	**Account for confounding with appropriate analysis**	
Fulde et al. 1995 [18]	Y	N	P	Y	N	13
A. Junge et al. 1995 [19]	Y	N	Y	Y	Y	19
Schade et al. 1999 [21]	Y	Y	Y	Y	Y	19
V. GRAVER et al. 1999 [20]	Y	P	P	Y	P	19
Kohlboek et al. 2004 [23]	Y	P	P	P	Not clear	14
L. Arpino et al. 2004 [22]	Y	Y	P	Y	P	15
Den Boer et al. 2006 [24]	Y	Y	Y	Y	Y	21
Silverplats et al. 2010 [27]	Y	Y	Y	Y	Y	19
JOHANSSON et al. 2010 [26]	Y	Y	P	Y	P	20
D’Angelo et al. 2010 [25]	Y	Y	P	Y	P	19
Kleinstueck et al. 2011 [29]	Y	Y	Y	Y	Y	19
Chaichana et al. 2011 [28]	Y	Y	Y	Y	Y	21
Sorensen and Mors 1989 [17]	Y	N	Y	P	P	16

### FAM Predictors

Different studies used different measures to capture FAM factors. Frequently used FAM measures were the McGill questionnaire and visual analog scale (VAS) for pain, the Tampa Scale for Kinesiophobia (TSK) [31] for fear-avoidance beliefs, the Roland Morris Disability Questionnaire for disability, the Zung Depression Scale (ZDS) [32], and the Beck Depression Inventory (BDI) [33] for depression.

### Pain

Seven studies examined pain, of which three (high quality studies) measured back pain and leg pain independently to predict LDH surgical outcomes [19,27,29]. In general, pain was always associated with LDH postoperative outcomes. When used independently, however, leg pain and back pain seemed to have different prognostic values. Patients with higher baseline leg pain had better surgical outcomes [19,27]. On the other hand, higher baseline back pain predicted worse outcomes [19,29].

### Catastrophizing, coping, anxiety, and fear-avoidance

Four studies examined pain coping or pain catastrophizing, four examined anxiety, and four studies examined fear and avoidance beliefs. Two (one high and one low quality study) of the four studies that measured pain coping preoperatively reported association with postoperative outcomes [18,24]. The two studies (one high and one low quality studies) that measured anxiety found an association with LDH surgical outcomes [17,25]. Three (high quality studies) out of four studies that measured fear and avoidance beliefs found an association with LDH surgical outcomes [19,24,26].

### Physical activity (PA), disability, and depression

Among all included studies in this review, PA level was measured in only one study (high quality study) [26]. PA level was addressed through a question; and this study did not report PA level to be associated with LDH surgical outcome. Functional disability was examined preoperatively in five studies. Three studies, all high quality, found disability to be associated with surgical outcomes [19,21,24]. The most measured FAM factor in the included studies was depression. Seven (five high quality) out of 10 studies that measured depression preoperatively found it to be associated with LDH outcomes [17,19,21-23,27,28].

## Discussion

Our aim was to systematically review prospective studies that examined preoperative FAM factors to predict LDH surgical outcomes. It was not our purpose to examine the psychometric properties of various instruments and therefore some differences in the results could be due to different measurement tools. We found 13 studies that fit our inclusion criteria. Most of these studies were considered of high methodological quality level except four. In general, many preoperative FAM measures were associated with LDH postoperative outcomes. In fact, some results indicate that psychological factors may have stronger association with outcomes than biomedical factors and these findings are in agreement with previous research that have included nonoperative patients with nonspecific LBP [8,34,35]. Overall, LDH outcome appears to be dependent on what outcome measure is used and many of these measures appear to be related to FAM factors.

Many studies used leg pain and back pain interchangeably to predict outcomes. However, studies that evaluated these two variables separately found leg pain and back pain to have different prognostic values. Patients with high leg pain but with less back pain had better outcomes. Fear and avoidance behaviors were measured in four studies, three of them found association with LDH surgical outcomes. The TSK was used to measure fear and avoidance beliefs in two studies. Fear and aviodance beliefs measured with the TSK was a predictor for LDH postoperative pain [24], disability [24], and quality of life [26] outcomes. Pain catastrophizing and physical activity level were the least studied FAM factors in relation to LDH surgical outcomes. Depression was the most commonly examined FAM factor, measured in 10 studies, of which 7 found baseline depression to be associated with LDH postoperative outcomes. Frequently used depression measures were the ZDS and BDI.

There was clear heterogeneity among the included studies in many aspects. Studies included in this review differed in the specific FAM measures employed, the statistical analyses performed, the variables used as covariates in the prediction models, and the outcome measures used. Moreover, sample sizes and follow-up periods varied considerably. Therefore, the results of this systematic review should be interpreted carefully considering each individual study’s predictors, outcomes, and results. Although we considered most of the related databases, one limitation of this systematic review could be that we missed potential studies in other databases.

A number of systematic reviews have looked at the relationship between psychological factors and postsurgical outcomes. Hinrichs*-*Rocker et al. [13] reviewed studies that examined the association between psychosocial factors and chronic postsurgical pain. Although this systematic review has included surgical procedures other than spine surgery, the finding showed likely associations between depression, psychological vulnerability, stress and chronic postsurgical pain. Celestine et al. [15] systematicly reviewed studies that assessed the relationship between presurgical pain, psychological, fuction variables and spine postsurgical outcome. While this review included patients who had undergone discectomy surgery, it also included patients who had undergone other spine procedures (e.g., fusion). In general, a positive relationship was found between psychological factors and poor surgical outcomes. Den Boer et al. [14] included only studies involving patients undergoing LDH surgery. They examined the predictive value of psychosocial and other factors with regard to post surgical outcome. Preoperative pain, disabiity, anxiety, somatization, and passive coping strategies predicted LDH postoperative outcomes. The search included studies that were done between 1980 and 2003. A number of related studies have been done since that time and we were able to include these studies. Our findings were in line with previous reviews and add additional insight on the influence of FAM factors on LDH postsurgical outcome.

## Conclusions

Although few studies were included in this review, this is the first systematic review that looked at the influence of FAM factors, exclusivily, on LDH postsurgical outcome. FAM factors appear to impact surgical outcomes on patients with LDH. Future prospective studies should confirm these findings and examine the prognostic value of more FAM measures in patients with operative and specific LBP cases. Pain catastrophizing and physical activity should be examined more in future studies because they have been found to be associated with oucomes in patients wih nonspecific LBP, but have been rarely examined in postoperative patients. Patients’ selection for conservative or operative management should take into account leg pain as well as back pain, depression and fear-avoidance beliefs.

## Abbreviations

LDH: Lumbar disc herniation; FAM: The fear avoidance model; LBP: Low back pain; FU: Follow-up; QA: Quality assessment; VAS: The visual analog scale; TSK: The tampa scale for kinesiophobia; ZDS: The zung depression scale; BDI: The beck depression index; PA: Physical activity.

## Competing interests

The authors declare that there are no competing interests.

## Authors’ contributions

FA and JF designed the research and the protocol; FA and KM conducted the search, assessed the potential studies, and conducted the quality assessment of the included studies; JF has served as a third reviewer in case of disagreement; FA extracted the data and wrote the paper. All authors read and approved the final manuscript.

## Supplementary Material

Additional file 1: Table S4 Summary Table A of the Included Studies (Aim, Sittings, Sample, Follow-up, and Baseline Measures). Click here for file

Additional file 2: Table S5 Summary Table B of the Included Studies (predictors, outcomes, analysis, results, findings, and comments). Click here for file
