# Supplementary material for: Do preoperative fear avoidance model factors predict outcomes after lumbar disc herniation surgery? A systematic review
Source: Chiropr Man Therap. 2013 Nov 18;21:40. doi: 10.1186/2045-709X-21-40 (PMC4176980; doi:10.1186/2045-709X-21-40)
Supplement: Additional file 1: Table S4 — Summary Table A of the Included Studies (Aim, Sittings, Sample, Follow-up, and Baseline Measures). [file 2045-709X-21-40-S1.docx]

**Table 4 - Summary Table A of the Included Studies (Aim, Sittings, Sample, Follow-up, and Baseline Measures)**

| **Study** | **Aim** | **Sittings (date, location, surgery)** | **Sample (size, age, gender, criteria)** | **Follow-up (size at FU)** | **Baseline pain or disability** |
| --- | --- | --- | --- | --- | --- |
| Sorensen and Mors 1989 | To test the prognostic value of social, psychological, and medical factors for postsurgical outcome. | Between January 1, 1985 to November 30, 1985  Department of Neurosurgery at the University Hospital, Aarhus, Denmark  Surgical procedure:  Discectomy | 57 patients  Neither age nor sex was reported?  Inclusion criteria: surgery was indicated according to the usual principles of the department and the patient's acceptance of participation in the study.  Exclusion criteria: previous operation for lumbar disk herniation and/or acute indication for operation. | 6 and 24 months FU  1 died in the 24-month FU | No report of baseline measures except that (72% of the patients = VAS>50mm) |

| **Study** | **Aim** | **Sittings (date, location, surgery)** | **Sample (size, age, gender, criteria)** | **Follow-up (size at FU)** | **Baseline pain or disability** |
| --- | --- | --- | --- | --- | --- |
| Fulde et al. 1995 | To investigate the predictive usefulness of defense mechanisms, coping strategies, and depression, for poor operation outcome | No dates reported  Department of Neurosurgery at the Endoklinik, Hamburg, Germany  Surgical procedure: discectomy | 52 consecutive patients (28 men)  Mean age = 41.4 (*SD* = 11.5)  Inclusion Criteria: Diagnosed with LDH and undergoing surgery | 6 months  48 patients (92%) completed the 6 months FU | Not reported |

| **Study** | **Aim** | **Sittings (date, location, surgery)** | **Sample (size, age, gender, criteria)** | **Follow-up (size at FU)** | **Baseline pain or disability** |
| --- | --- | --- | --- | --- | --- |
| A. Junge et al. 1995 | To determine which of the somatic subjective symptoms, objective signs, sociodemographic, and psychological factors influence the outcome of lumbar disc surgery, and to develop screening checklist to distinguish bad and good surgery outcome. | No dates were reported  6 centers in Germany and Switzerland (Neurosurgery and orthopedic surgery departments)    Surgical procedure: Standard discectomy | 400 consecutive patients (19 were reoperated and excluded)  381 patients (229 men)  Mean age = 44.7 (*SD* =11.2 y/o)  Inclusion Criteria: Age less than 69 years, German as a native language, Indication for LDH surgery, and no previous disc or back surgery. | 6 and 12 months FU  342 (89%) completed the 6 months FU  328 (86%) completed the 12 months FU | Mean pain (VAS) = 6.5 (*SD* = 2.87  BDI = 7.46 (*SD* = 5.72) |

| **Study** | **Aim** | **Sittings (date, location, surgery)** | **Sample (size, age, gender, criteria)** | **Follow-up (size at FU)** | **Baseline pain or disability** |
| --- | --- | --- | --- | --- | --- |
| Schade et al. 1999 | To investigate the predictive value of three classes of variables (medical data including demographics, low-back pain history, physical findings and MRI-identified morphological abnormalities, general psychological factors and psychosocial aspects of work) for discectomy outcome. | March 1991 to Oct 1993 (original study: Boos et al. 1995)  Sittings was not reported  Surgical procedure: discectomy | 46 consecutive patients (34 men) with a symptomatic LDH  35.2 y/o (range 20-50) (Boos et al. 1995)  Inclusion Criteria: a scheduled discectomy, age 20–50 years, continued employment at the time of surgery, no previous back surgery, failed adequate trial of conservative treatment (6–8 weeks), and availability for an additional clinical and MRI examination prior to the surgery  Exclusion criteria: not Swiss residentship, rapid progressive severe motor deficit or cauda equina. | 2 year FU (23-30 months)  42 of the 46 patients (91.3%) completed the 2 year follow-up | Presented in bar chart |

| **Study** | **Aim** | **Sittings (date, location, surgery)** | **Sample (size, age, gender, criteria)** | **Follow-up (size at FU)** | **Baseline pain or disability** |
| --- | --- | --- | --- | --- | --- |
| V.GRAVER et al. 1999 | To evaluate the long-term (7 years) results, and the predictive value of general background variables, psychological traits, fibrinolytic activity for surgical outcome.  In addition, To reassess psychological traits and relate the findings to the long-term clinical outcome. | From August 1988 until March 1990  All operations were at the Department of Neurosurgery, Ullevaal University Hospital, Norway.    Surgical procedure: discectomy | 122 consecutive patients (56 women)  7-year follow-up  🡪114 (93%)  Mean age = 40.5 y/o  96 (42 women) attended the clinical evaluation (constitute the main sample in the study)  18 other patients (12 women) only mailed the questionnaire  Inclusion Criteria: clinical symptoms and corresponding neuroradiological findings of nerve root compression due to lumbar disc herniation, with little or no associated osteodegenerative changes  Ex criteria: other diseases of the lumbosacral spine, previous low back surgery, and over 70 y/o. | 1 and 7 year (range 6- 7.5 years) follow-up  7-year follow-up  🡪114 (93%) | LBP = 55.6 (*SD* = 24.1)  LP = 61.5 (*SD* = 22.8) |

| **Study** | **Aim** | **Sittings (date, location, surgery)** | **Sample (size, age, gender, criteria)** | **Follow-up (size at FU)** | **Baseline pain or disability** |
| --- | --- | --- | --- | --- | --- |
| Kohlboek et al. 2004 | To assess the prognostic power of somatic, psychologic, and social predictors for the 6-month outcome of lumbar discectomy. | Between July 2001 and February 2002  Neurosurgery Department of Salzburg, Austria    Surgical procedure: microdiscectomy | 58 consecutive patients  Mean age = 47 years (*SD* = 11.73)  Inclusion criteria: 1) clinical symptoms, corresponding neurodiagnostic findings and pain duration of more than 6 weeks; 2) no red flags (serious spinal pathology) or other diseases of the lumbosacral spine (e.g. instability, spondylolisthesis); 3) no previous back surgery; 4) German as native language; and 5) age less than 70 years. Six patients were excluded due to acute surgery, and another four patients refused to participate in this study. | 6 months follow-up  48 patients (83%) (29 men) completed the 6 months follow-up | Neither preoperative pain, nor disability score were reported.  Mean duration of pain was 10-weeks (*SD* = 15.12), and patients reported an average number of similar pain episodes of 3.37 (*SD* = 6.50). |

| **Study** | **Aim** | **Sittings (date, location, surgery)** | **Sample (size, age, gender, criteria)** | **Follow-up (size at FU)** | **Baseline pain or disability** |
| --- | --- | --- | --- | --- | --- |
| L. Arpino et al. 2004 | To examine the role of depressive condition in the outcome after LDH surgery. | Between Sept 2001 and may 2002  Neurosurgery Dept. at the University of Naples Italy    Surgical procedure: microdiscectomy | 73 (25 women) consecutive patients  Mean age of 43.5 y/o *SD* = 15.3 y  Inclusion criteria (no criteria were reported but just this 2 statements): LDH candidates. Diagnosis was based on clinical and neuroradiological evaluations including lumbar spine radiography and MRI. | 3 and 12 months post operatively  FU sample size was not reported | Baseline pain = 6.4 (range = 1.4–10.0) |

| **Study** | **Aim** | **Sittings (date, location, surgery)** | **Sample (size, age, gender, criteria)** | **Follow-up (size at FU)** | **Baseline pain or disability** |
| --- | --- | --- | --- | --- | --- |
| Den Boer et al. 2006 | To clarify the role of preoperatively assessed cognitive-behavioral factors ((i.e., pain related fear of movement/ (re) injury, passive pain coping, and negative outcome expectancies) in postop disability and pain-intensity in patients who underwent surgery for LDH. | No reported dates  Four Dutch hospitals (their names reported in the study)  Surgical procedure: Standard discectomy | 310 patients  277 had complete data, 50% were female  Mean age = 43 (range 17-77)  Inclusion Criteria: First time lumbar disc surgery, age older than 16 years, failure of conservative treatment, and an ability to understand and read  Dutch  Exclusion criteria: co-morbidity influencing daily activities and. | 6 weeks and 6 months postop  277 (89%) had complete data | VAS pain = 47.3 (*SD* = 21.6)  Ronald disability = 15.3/24 (*SD* = 4.1) |

| **Study** | **Aim** | **Sittings (date, location, surgery)** | **Sample (size, age, gender, criteria)** | **Follow-up (size at FU)** | **Baseline pain or disability** |
| --- | --- | --- | --- | --- | --- |
| Silverplats et al. 2010 | To examine the long term outcome of lumbar disc herniation surgery and to investigate if any demographics, psychological, social or physiological factors could predict the surgical outcome. | Between September 1996 and March 2002  Location was not reported in the study but in the author info-Sweden)  Surgical procedure: discectomy or microdiscectomy | 183 consecutive patients  171 patients met inclusion criteria  Mean age of 39, *SD* = 11 years    76 (44%) of the patients were women  Inclusion Criteria: first time surgically treated patients with a CT or MRI-verified one-level disc herniation on L4–L5 or L5–S1 level that correlated with the patients’ symptoms  Exclusion criteria: Patients with previous surgery on the herniated disc segment or with other spinal disorders | 2 year and long follow-up (mean = 7.3, *SD* = 1.0) (Range = 5.1 – 9.3 years)  154 (90%) at 2-year follow-up  140 (81%) at long term follow-up | VAS leg pain Mean = 59, *SD* = 19  VAS back pain  Mean = 50, *SD* = 23  ODI mean = 53 |

| **Study** | **Aim** | **Sittings (date, location, surgery)** | **Sample (size, age, gender, criteria)** | **Follow-up (size at FU)** | **Baseline pain or disability** |
| --- | --- | --- | --- | --- | --- |
| Johansson et al. 2010 | To analyze the predictive value of cognitive and behavioral factors for pain, disability and quality of life 1 year after lumbar disc surgery. | Between March 2003 and March 2005  Two orthopedic departments in Sweden, one university department (*n*=41) and one community hospital (*n*=18)  Surgical procedure: Standard discectomy | Consecutive patients  59 patients met inclusion criteria  Mean age = 40, *SD* = 8 y/o, 40% female  In Criteria: First time lumbar disc surgery, 18 to 60 y/o, MRI-confirmed lumbar disc herniation  Ex criteria: co-morbidity influencing daily activities and not being fluent in the Swedish language. | 1 year  55 (93%) at 1year follow-up | Median baseline:  Leg pain (0–100) = 72  Back pain (0–100) = 70  ODI-Swedish version (0-100) = 38 |

| **Study** | **Aim** | **Sittings (date, location, surgery)** | **Sample (size, age, gender, criteria)** | **Follow-up (size at FU)** | **Baseline pain or disability** |
| --- | --- | --- | --- | --- | --- |
| D’Angelo et al. 2010 | To evaluate anxiety and depression as prognostic factors for radicular and back pain after 1 year postop in patients with lumbar disc herniation. | Between April 2006 and November 2007  Study took place in Neurosurgery Department of “Casa Sollievo della Sofferenza” Hospital, San Giovanni Rotondo (Italy)    Surgical procedure: microdiscectomy | 142 patients met criteria, 108 included in the statistical analysis (64 men)  Mean age=45.9, *SD* = 12.2 y/o  Inclusion Criteria: LDH and persistent radicular pain with or without LBP despite nonsurgical treatment for at least 6 weeks, evidence of nerve root irritation with a positive Lasègue sign and/or a corresponding neurological deficit. Neuroradiological examination with confirmed disc herniation at a level and side corresponding to the clinical symptoms  Exclusion criteria: younger than 18 years old, previous lumbar surgery, cauda equine syndrome, or other diseases of the lumbosacral spine, pregnancy, or preexisting psychiatric diseases | 1, 3, 6, 12-month follow-up  108 patients completed the 12 month measures and included in the statistical analysis (64 men)  25 (17.6%) did not adhere to the psychometric evaluation during the 12-month study period and were excluded | VAS mean = 8  ZDS mean = 12 |

| **Study** | **Aim** | **Sittings (date, location, surgery)** | **Sample (size, age, gender, criteria)** | **Follow-up (size at FU)** | **Baseline pain or disability** |
| --- | --- | --- | --- | --- | --- |
| Kleinstueck et al. 2011 | To examine how the relative severity of LBP influences the outcome of lumbar decompression surgery for lumbar disc herniation. | Between March 2004 to April 2008  The data were taken using the framework of the Spine  Society of Europe (SSE) Spine Tango Spine Surgery  Registry together with the author local spine surgery outcomes database    Surgical procedure:  Standard discectomy | Consecutive patients  308 patients met criteria (177 men)  Mean age= 48, *SD* = 13  In Criteria: The patients had to have a good understanding of written German or English or (after 2006) French, Spanish, Italian or Portuguese, have a 1-year follow-up questionnaire, and satisfy the study’s surgical admission criteria (single level LDH no additional pathology, posterior decompression by means of discectomy or sequestrectomy, with no additional fusion or stabilization) | 12 months  92% of patients who were sent the 12-month completed the 12 months FU  “Although all 308 patients had a 12-month questionnaire,  46 of them had no baseline questionnaire due to administrative errors (*n* = 5) or because the patient was admitted on an emergency basis (*n* = 36)” | Baseline leg pain = 6.9, *SD* = 2.5  Back pain = 4.4, *SD* = 3.0  Main problem: back pain (14.6%)  Leg pain (55.1%)  Neurological disturbances (30.3%) |

| **Study** | **Aim** | **Sittings (date, location, surgery)** | **Sample (size, age, gender, criteria)** | **Follow-up (size at FU)** | **Baseline pain or disability** |
| --- | --- | --- | --- | --- | --- |
| Chaichana et al. 2011 | 1- To determine the role that preoperative depression and somatic anxiety have on long-term back and leg pain, disability, and quality of life (QOL) for patients undergoing single-level lumbar discectomy.  2- To assess whether depression and somatic measures were associated with the achievement of an MCID in all outcome measures. | No dates reported  2 medical institutions    Surgical procedure: microdiscectomy | 67 patients (42 men)  Mean age = 41+-10 y/o  Inclusion Criteria: diagnosis of sciatica or persistent LBP, failed 6-week minimum of conservative therapy, and neurological deficit. MRI-confirmed LDH corresponding with patient symptoms.  Exclusion criteria: previous back surgeries, multilevel disc herniation, foraminal or extraforaminal herniation, extraspinal cause of sciatica, active medical or workers’ compensation lawsuit, preexisting spinal pathology, unwilling to participate in follow-up procedures, notable nonintervertebral disc abnormalities including spondylolysis, spondylolisthesis, inflammatory arthritis, or metabolic bone disease, or chronic back pain unrelated to their recent development of LDH | 3, 6, 9, and 12 months after surgery  1-year no loss to FU (*n* = 67) | Preop VAS-BP mean = 6.1, *SD* = 5.6  VAS-LP mean = 6.1, *SD* = 5.6  ODI mean = 49.9, *SD* = 17.6  ZDS mean = 18.5, *SD* = 10.6 |
