# Supplementary material for: Do preoperative fear avoidance model factors predict outcomes after lumbar disc herniation surgery? A systematic review
Source: Chiropr Man Therap. 2013 Nov 18;21:40. doi: 10.1186/2045-709X-21-40 (PMC4176980; doi:10.1186/2045-709X-21-40)
Supplement: Additional file 2: Table S5 — Summary Table B of the Included Studies (predictors, outcomes, analysis, results, findings, and comments). [file 2045-709X-21-40-S2.docx]

**Table 5 - Summary Table B of the Included Studies (predictors, outcomes, analysis, results, findings, and comments)**

| **Study** | **Predictors** | **Outcome** | **Analysis** | **Results/findings-comments** |
| --- | --- | --- | --- | --- |
| Sorensen and Mors 1989 | Pain (VAS)  Pain drawing (rating chart)  Minnesota Multiple Personality  Inventory (MMPI) including Depression scale  Sex  Age  Employed  Duration of sick leave  Duration of back illness  Duration of education  Social support  Myelography and surgical findings  Rahe’s and Holme’s Life events (short version) | Poor or good operation outcome  Poor = state of health about the back "poor" (patients stated their health about the back as poor, fair or quite good), pain drawing on VAS >= 50, and no job function (not employed).  Every operation outcome other than "'poor surgical outcome'" is defined as good surgical outcome  The total surgical outcome of each patient from the 2 follow-up stated as:  2 = good outcome both 6 and 24 months postoperative.  1 = poor outcome either 6 or 24 months postoperative.  0 = poor outcome on both FU | Multiple linear regression (Variables were entered to the regression analysis one by one, and only withheld a variable if it increased the regression coefficient significantly with *p* < 0.05) | At the 24-month follow up, 25% of patients reported that there back health is “poor”  In the univariate analysis, pain, pain drawing, anxiety and depression (MMPI) were found significant predictors.  Main finding of the multivariate regression results were: Ad-scale (MMPI) and being employed or not, together explained 42% of the difference in surgical outcome.  None of the included FAM variables were entered in the multiple regression analysis. Variables were entered to the regression analysis one by one, and only withheld a variable if it increased the regression coefficient significantly with *p* < .05 |

| **Study** | **Predictors** | **Outcome** | **Analysis** | **Results/findings-comments** |
| --- | --- | --- | --- | --- |
| Fulde et al. 1995 | Beck Depression Inventory (BDI)  Coping strategies questionnaire consisted of 5 scales (Avoidance of movement, other avoidance, search for social support, distraction, and nonverbal pain expression).  Defense mechanism questionnaire consisted of 5 scales (renationalization, denial, turning against the object, regression, avoidance of social contact). | Poor operation outcome (All 3 criteria: use of analgesics because of pain, frequent visits to the doctor because of pain, and failure to return to work). | Stepwise discriminant analysis (5 coping items + 5 defense items + 1 depression item have been used in this analysis). | 8 patients (16,7%) matched all three “poor” outcome criteria.  5 out of 6 items (regression, rationalization, avoidance of social contact, nonverbal pain expression, search for social support) correctly predicted 87.5% of the poor surgical outcome and 80% of the good surgical outcome group (2 from the coping strategies and 3 from the defense mechanisms).  39/48 (81.3%) of the patients’ outcomes were correctly predicted using the 5 variables found relevant.  Depression was not found to be predictive of the poor outcome. The BDI did not correlate with any other variable at baseline. |

| **Study** | **Predictors** | **Outcome** | **Analysis** | **Results/findings-comments** |
| --- | --- | --- | --- | --- |
| A. Junge et al. 1995 | Hannover mobility questionnaire (HMQ)  Beck depression questionnaire (BDQ)  Pain behavior (5 scales: search for social support, avoidance of movement, general avoidance, distraction/relaxing, nonverbal expression)  Sociodemographic:  Gender  Job level  Desire for disability pension  Duration of reduced working ability  Duration of acute back pain  Intensity of LBP (VAS)  Suffering from complaints  Number of other pain location  **Predictors**  (Continuous) | 12-month outcome  1- LBP >= 6 on VAS and  2- Reduced working ability of more than half a year or no return to previous job and  3- Regular visit to the treating physician or hospital stay  Bad **=** 2 criteria if back pain was 4; or all 3 criteria  **Outcome**  (Continuous) | Multivariate canonical discriminate analysis of the 12-months outcome (good-bad)  **Analysis**  (Continuous) | At the 12-months FU, 169 (51.5%) were in the good outcome group, 93 (28.4%) in the moderate outcome group, and 66 (20.1%) in the bad outcome group  No significance differences in outcome between 6 and 12 months FU  About 80% of the patients were classified correctly by the statistical model  Pain history (physical mobility, duration of reduced working ability and of acute back pain, intensity of LBP, suffering from complaints, number of other pain location contributed to correct outcome 73.8%  Psychodiagnostic parameters (search for social support, avoidance of movement, distraction, other pain behavior and depression) contributed to correct outcome 62.9%  Medical parameters (pain radiation to leg, additional back Dx, imaging finding, and SLR) contributed to correct outcome 57.7%  **Results/findings-comments**  (Continuous) |
| A. Junge et al. 1995 | Pain radiation to leg  Additional back Diagnoses  Imaging finding  SLR | Moderate = one of the criteria above; or 2 if LBP is between 0 and 3  Good outcome = none of the abovementioned criteria |  | The strongest predictors were physical mobility (HQM), number of pain location in the body before surgery, duration of reduced working ability, duration of acute back pain, and suffering from complaints (VAS). Low job level, HPQ (search for social support), and intensity of acute back pain were also predictive of bad outcome.  The study found that patients with no reduced mobility, no back pain, and no other painful spots in the body but with disabling leg pain with radicular tension signs and differences in jerk reflexes and/or muscular palsy had the best surgical outcome. Those with long-lasting preoperative working disability, primary back pain, other painful sources in the body, and low job level and low education level were found more likely to have bad surgery outcome.  Depression and pain behavior are important predictors if the postoperative outcome was only pain. However, psychological factors lost their importance when all 3-outcome criteria considered. |

| **Study** | **Predictors** | **Outcome** | **Analysis** | **Results/findings-comments** |
| --- | --- | --- | --- | --- |
| Schade et al. 1999 | Medical data (including demographics,  LBP history, physical findings, MRI-identified morphological abnormalities)  Psychological general well-being index (Depression, Anxiety, self-control, well-being, general health, and vitality)  Psychosocial factors (Job satisfaction and social support  Preoperative pain (McGill pain questionnaire, VAS)  (Control variable)  Preoperative disability (RMDQ)  (Control variable) | Low Postop pain (VAS)  Disability (Ronald Morris)  Return to work (Time in months)  Surgical outcome (4 items about pain, work, medication, and physical limitations) | Stepwise multiple regression analyses performed separately for each of the three variable sets (medical data, general psychological factors and psychosocial aspects of work).  Then the significant variables of the three categories were combined in an overall regression model with ‘preoperative pain and/or preoperative disability in daily activities’ entered first in the regression model. Then, medical data were entered followed by general psychological variables, and work- | The final regression model to predict pain relief demonstrated MRI identified neural compromise (.28, *p* < .1) and social support by the spouse (-.39, p < .01) (and control variable preop pain, -.42, *p* <.01) as significant predictors of pain relief at 2 year follow-up (adj *R^2^* = .3, *p*<.01).  The final regression model to predict disability showed that MRI-identified neural compromise (-.44, *p* < .05) and work-related resignation (.4, *p* < .001) (and control variable preop disability, .39, *p* < .05) were significant predictors of subjective disability at 2 year follow-up (adj *R^2^* = .46, *p* <.0001).  The final regression model to predict return to work showed that depression (.43, *p* < ,01) and occupational mental stress (.28, *p* < .01) (and control variable preop pain disability, .35, *p* < .05) were significant predictors of return to work at 2-year follow-up (adj *R^2^*=.31, *p*<.001).  The final regression model to predict surgical outcome showed that the extent of herniation (.48, *p* < .001) and depression (-.46, *p* < .001) (and control variable preop pain/disability, -.46, *p* < .01) were significant predictors of a good result after lumbar discectomy. |
|  | **Predictors**  (Continuous) | **Outcome**  (Continuous) | **Analysis**  (Continuous) | **Results/findings-comments**  (Continuous) |
| Schade et al. 1999 |  |  | related psychosocial factors. | Among all FAM factors in this study, preoperative pain, disability, and depression showed significant prediction of postop pain, disability, or RTW. |

| **Study** | **Predictors** | **Outcome** | **Analysis** | **Results/findings-comments** |
| --- | --- | --- | --- | --- |
| V.Graver et al. 1999 | Psychological traits:  Modified Somatic Perception Questionnaire (MSPQ) and Hospital Anxiety and Depression Scale (HAD)  Fibrinolytic activity  Perioperative variables (One vs. two operated discs)  (Partial vs. full laminectomy  Background variables were controlled for (age, BMI, smoking, and alcohol consumption) | Clinical overall score (COS): from 96 patients, preop and 1 year postop:  1-pain (average VAS)  2-physical signs (e.g., SLR)  3-functional (ODI)  4-type and dosage of analgesics registered    Low back pain (VAS)  Leg pain (VAS) | Four multivariate regression analyses:  7-year outcome (COS, LBP, and leg pain)  Controlling for background variables (age, BMI, smoking, and alcohol consumption) | Gender (being female) significantly predicted poor COS, low back and leg pain  MSPQ, but not HAD, was significantly predictive of COS, low back and leg pain  The 7-year outcome was worse than the 1-year outcome for leg pain, back pain and disability and just back pain was significantly worse.  The 7-year psychometrical scores were significantly associated with the 7-year COS, HAD-anxiety (*F* = 24.80, *p* < .001, *R^2^* = 0.21), HAD-depression (*F* = 31.79, *p* < .001, *R^2^* = 0.26) and MSPQ (*F* = 49.89, *p* < .001, *R^2^* = 0.35).  6% had reoperation, 23% did not RTW, and 12% were partially satisfied or not satisfied 7 years postop |

| **Study** | **Predictors** | **Outcome** | **Analysis** | **Results/findings-comments** |
| --- | --- | --- | --- | --- |
| Kohlboek et al. 2004 | Medical factors (SLR, duration of pain, and radicular distribution of pain)  Psychological factors:  1-Pain (NPRS from 1 to 7) 2-Depression (ADS-L) 3-pain interference with ADL Pain disability index (PDI) 4-Qualitative assessment of pain (McGill pain Q) 5-Job strain (1-10). 6-Coping strategies (KSI)  Sociodemographic variables: (educational and social status, occupational characteristics, and duration of inability to work) | Pain maintenance: (one question about current pain; yes/ no)  Number of pain location (11 body regions)  Pain intensity (NPRS 1-10)  Functional status (Hannover Mobility Questionnaire (HQM)  Return to work (Y/N)  Health-Related Quality of life (MOS SF-36) | The six outcome criteria were classified into one outcome variable by hierarchical cluster analysis (Ward**’** s method) | 6 months postop outcome clustering yields 3 groups (success-14 patients, socially unintegrated-14 patients, and poor outcome group-21 patients)  44% (21) of the patients did not benefit from surgery when subjective outcome criteria were considered. This group had 5/10 NPRS 6 months postop.  SLR (the only significant one among medical factors), depression and sensory pain description (from the psychological variables) were significant in predicting the 6 months outcome group and all together classified 83% of outcome group.  Depression was the most important predictor among the psychological measures.  The greater the degree of preoperative depression, the worse the 6-month postoperative outcome.  The combination of medical, psychological, and social variables yielded the best prediction of the outcome classification.  SLR correctly classified 40% of outcome group  Depression correctly classified 48% of outcome group. |

| **Study** | **Predictors** | **Outcome** | **Analysis** | **Results/findings-comments** |
| --- | --- | --- | --- | --- |
| L. Arpino et al. 2004 | Depression (ZDS)  Age, sex, level of disc herniation were controlled for | Pain (VAS) at 12 months | Multiple regression analysis | No sig differences in outcome between 6 and 12 FU.  Depression was the only significant predictor (none of the controls) for the 12 months postop pain (*t* = 7.120, *p* < .001).  About half of the patients (36) were depressed (ZDS >= 35) preoperatively.  4 patients developed depression 3 and 12 months after surgery.  28/36 of the patients stayed depressed pre- and 12 months postoperatively.  After controlling of patients with absolute depression, patients with relevant depressive Symptoms (*n* = 4) had higher VAS at baseline (*F* = 6.7, *p* < 0.02), 3 (*F* = 179.6, *p* < .001), and 12 months postoperatively (*F* = 219.2; *p* < .001). (ANOVA analysis). |

| **Study** | **Predictors** | **Outcome** | **Analysis** | **Results/findings-comments** |
| --- | --- | --- | --- | --- |
| Den Boer et al. 2006 | Entered in the 3rd step in regression:  Fear of movement (TSK-AV-adjusted version) taken 1 day preop)  Passive pain coping (PCI taken 1 day preop)  Negative expectancy (4 item-scale taken 1 day preop)  Adjusted variables were entered in the 1st step in regression:  Disability (RDQ) –Dutch version  Preop Pain (VAS)  Demographics (age, gender, education level)  Entered in the 2nd step in regression:  **Predictors**  (Continuous) | Disability (RDQ)  Pain (VAS)  **Outcome** (Continuous) | Multiple regression analyses were used to study the contribution of cognitive-behavioral factors, after controlling for preop disability, preop pain, age, gender, and educational level (entered at step 1) and pain 3 days postop (entered at step 2).  **Analysis**  (Continuous) | Improvement after surgery was less obvious 6 months than 6 weeks. Significant difference in disability but not pain.  6 months postop, 31% of the patients still experienced high level of disability (RDQ >= 8/24) and 25% of severe pain (VAS >= 30/100).  Independent predictors of high disability at 6 weeks and 6 months postop were negative outcome expectancies (*t* = 2.62, *p* < .01; *t* = 3.25, *p* < .01), more pain-related fear of movement/(re) injury (*t* = 3.15, *p* < .01; *t* = 3.14, *p* < .01), and passive pain-coping strategies (*t* = 2.4, *p* <.05; *t* = 3.49, *p*< .01).  Independent predictors of higher pain at 6 weeks and 6 months postop were more negative outcome expectancies (*t* = 3.16, *p* < .01, *t* = 4.05, *p* < .001), more pain-related fear of movement/(re) injury (*t* = 2.92, *p* < .01; *t* = 2.07, *p* < .05), and passive pain-coping (*t* = 2.19, *p* < .05; *t* = 2.62, *p* < .01).  Higher levels of preop disability, preop pain, pain 3 days postop, negative outcome expectancies, fear of movement, passive pain-coping, older age, female gender, and lower level of significantly predicted more disability at follow-up assessment.  **Results/findings-comments**  (Continuous) |
| Den Boer et al. 2006 | 3-day postop pain (VAS) |  |  | 3-day postop pain (control variable) significantly predicted both pain and disability 6 weeks and 6 months postoperatively.  Higher levels of preop pain, pain 3 days postop, more preop neurological deficits, negative outcome expectancies, fear of movement, older age, and female gender, significantly predicted more pain intensity at follow-up assessment (6 weeks or 6 months).  Pain-coping strategies predicted 6-month disability but not future pain. |

| **Study** | **Predictors** | **Outcome** | **Analysis** | **Results/findings-comments** |
| --- | --- | --- | --- | --- |
| Silverplats et al. 2010 | Baseline back pain (VAS)  Baseline leg pain (VAS)  Duration of leg pain  Baseline depression (ZDS)  Baseline disability (ODI)  Gender, Age, Smoking habits, Level of disc hernia, Use of analgesics, Time on sick leave | Satisfaction with surgical treatment (primary dichotomized outcome)  Macnab postop classification (excellent/good or fair/poor) at 2 year F/U (primary dichotomized outcome)  Change in back pain, change in leg pain, working capacity, analgesics, sleeping pills (secondary outcomes at 2-year FU) | Logistic regression with all predictors that showed a potential influence in the bivariate analyses (i.e. predictors that showed a *p* < 0.20).  Logistic regression models were also analyzed with a forward (likelihood ratio) stepwise selection procedure, aiming at finding the most influential predictor. | 16 patients had undergone at least one re-operation on the lumbar spine.  33% had fair or poor on the primary on the primary objective outcome (2 year postop)  33% (2-year FU) 28% (7 year FU) were partially or not satisfied with surgical outcome.  Higher baseline leg pain was the only significant predictor of the improvement in postop leg pain (*p* < 0.039).  Depression was the only significant predictor of the improvement in back pain (*p* < 0.049).  The main finding of the study, and the predictor of the primary and many of the secondary outcomes, was length of sick leave.  Sick leave correlated with duration of leg pain, which might gave nonsignificant results for duration of leg pain.  Neither baseline back pain nor baseline disability was significant predictors of any of the surgical outcome. |

| **Study** | **Predictors** | **Outcome** | **Analysis** | **Results/findings-comments** |
| --- | --- | --- | --- | --- |
| Johansson et al. 2010 | Leg and back pain (VAS)  Fear avoidance behavior (TSK)  Coping/catastrophizing (CSQ)  Expectation to return to work 3 months after surgery  Age, gender, educational level, sick leave, work load, physical activity level, and duration of current leg and back pain | Leg and back pain (VAS)  Disability (ODI)  Quality of life (EQ-5D)  Sick leave | Multiple backward logistic regression to examine prognostic of preop fear-avoidance, coping, and expectation to RTW to dependent variables (pain, disability, and QOL).  Subsequent Multiple Logistic regression analysis included independent variables that fulfilled the multicollinearity restriction (correlation coefficient *r* < 0.4) and had p-value of < 0.1 in the first regression analysis.  Variables entered in the final regression model were age, gender, work load, duration of leg pain, coping catastrophising, fear avoidance beliefs and expectations of chance to RTW within 3 months after surgery.  Age, gender, educational level, workload, leg pain, and rehabilitation group were controlled for. | 9 (16%) patients remained on sick leave 12 months postop.  The strongest predictor for low quality of life 12 months after surgery was high scores of fear avoidance beliefs (OR = 6.6, *p* < 0.027).  Fear avoidance was not included as predictor of other outcomes.  Other FAM variables did not reach significance.  The main finding was that expectation to return to work within 3 months postop (one question: In your estimation, what are the chances that you will be working in 3 months?) was significant predictor for all outcomes.  Female was predictor of worse QOL after 1 year LDH surgery (OR = 6, *p* <0.03). |

| **Study** | **Predictors** | **Outcome** | **Analysis** | **Results/findings-comments** |
| --- | --- | --- | --- | --- |
| D’Angelo et al. 2010 | State (y1) and Trait (y2) Anxiety Inventory (STAI)  Depression (ZDS)  (All variables with *p* < .10 at univariate analysis were entered in the multiple linear regression) | Pain (VAS) | Multiple linear regression analysis  The variables with *p* < .10 from the univariate analysis were entered in the multiple linear regression analysis | 12 months postop, 38 (35%) reported only radicular leg pain, and 25 (23%) reported both radicular leg and back pain.  In the univariate analysis, the presence of preoperative pain was significantly correlated with state anxiety (*p* < .0001), trait anxiety (*p* < .0001), and inability to work (*p* < .0001).  From the regression analysis, preop trait anxiety was the main predictor of the severity of pain (VAS score) both before (*p* < .001) and after surgery (*p* < .001).  59 (54.6%) of patients showed trait anxiety before surgery, and 12 (11,1%) showed depression.  A significant increase (*p* < .004) in current depression (ZDS > 49/80) was found before and 12 months after surgery; From 12 (11%) to 25 (23%).  Depression was present in patients showing trait anxiety and persistent pain after surgery.  The persistence of pain during the follow-up was significantly associated with the presence of pain at each previous follow-up. (Regression analysis result). |

| **Study** | **Predictors** | **Outcome** | **Analysis** | **Results/findings-comments** |
| --- | --- | --- | --- | --- |
| Kleinstueck et al. 2011 | Entered in the 2nd step in regression:  LP  LBP  LP-LBP (LP intensity minus the preoperative LBP intensity)  **(**Leg/buttock and back pain intensity, each measured separately on a 0–10 graphic rating scale)  Adjusted variables were entered in the 1st step in regression:  Age, Gender, Comorbidity, Multidimensional Core Outcome Measures Index (COMI) questionnaire: questions about pain (leg/buttock and back pain intensity measured with 0-10 graphic rating scale), function, symptom specific well-being, general quality of life, and social and work disability. | COMI  Question about global outcome (GO) of surgery “how much did the operation help your back problem?”  (5-likert scale dichotomized into Good or poor) | Multivariate stepwise regression analysis was used to predict the 12-month postop COMI score. The preop COMI score, age, gender, and comorbidity were entered in the first step (adjusted variables), and in the 2^nd^ step: preop LP, LBP and LP-LBP (as potential predictors, using forward selection).  Multivariate stepwise logistic regression analysis to predict the 12-month postop GO of surgery (good or poor).  The baseline age, gender, and comorbidity were entered in the first step (adjusted variables), and in the 2^nd^ step: preop LP, LBP and LP-LBP (as potential predictors, using forward selection). | 12 months dichotomized GO🡪 249/308 (81.1%) patients had a ‘‘good’’ outcome, and 58/308 (18.9%) had a ‘‘poor’’ outcome.  In the multiple regression, After controlling of age, gender, comorbidity, and COMI, preop LBP were the most significant predictor of the 12-month COMI score (*b* = 0.204, *p* = .001).  Higher preop LBP predicted worse outcome (i.e., COMI).  In the multiple logistic regression, After controlling of age, gender, comorbidity, and COMI, baseline LBP was the only significant predictor of the 12-month the global outcome (OR = 0.821, *p* < .004).  Higher preop LBP predicted worse outcome (i.e., less chance of getting good outcome). |

| **Study** | **Predictors** | **Outcome** | **Analysis** | **Results/findings-comments** |
| --- | --- | --- | --- | --- |
| Chaichana et al. 2011 | Depression (ZDS)  Somatization (MSPQ)  (Controlling for patient age, weight, and preoperative VAS-BP, VAS-LP, ODI, or SF-36 PCS score) | VAS-BP  VAS-LP  ODI  SF-36 PCS (QOL)  MCID for each outcome | Regression analysis to predict the 1-year outcome.  Multiple linear regression analysis to examine the relationship between preoperative ZDS and  MSPQ scores and the 12-month improvement in VAS-BP, VAS-LP, ODI, and SF-36 PCS scores. (Controlling for patient age, weight, and preoperative VAS-BP, VAS-LP, ODI, or SF-36 PCS score).  Logistic regression analysis to examine the relationship between preoperative ZDS and MSPQ scores and the 12-month improvement in VAS-BP, VAS-LP, ODI, and SF-36 PCS that achieved the MCID. (Controlling for patient age, weight, and preoperative VAS-BP, VAS-LP, ODI, or SF-36 PCS score). | 10% (7 patients) were depressed preop (ZDS>33)  In the multivariate linear regression analysis, increase in preop ZDS score or MSPQ were associated with less improvement in VAS-BP score (p = .02, *p* = .005), less improvement in VAS-LP score (*p* = .03, p = .001), less improvement in ODI score (*p* < .001, *p* = .001), and less improvement in SF-36 PCS score (*p* < .001, *p* = .001).  In the multivariate logistic regression analysis, each 1-point increase in preop ZDS score or MSPQ were associated with (12%, 21%) and (11%, 13%) less likelihood of achieving an MCID in ODI (*p* = .006, *p* = .002) and SF-36 PCS (*p* = .04, *p* = .03) at 1 year postoperatively, respectively.  Neither the preop ZDS nor MSPQ were significant predictors of failure to reach an MCID in the VAS-BP (*p* = 0.85, *p* =.77) or VAS-LP (*p* = .96, *p* =.64) score.  The results suggest that depression and somatic awareness may not impact the amount of pain relief reported after discectomy, but rather impacted QOL and functional disability in the LDH patients. |
